# Excessive Internet use and depressive symptom levels in adolescents with depressive disorders: chain mediation of social anxiety and sleep quality

**DOI:** 10.3389/fpsyt.2026.1799801

**Published:** 2026-05-04

**Authors:** Mengyuan Zhang, Liqi Gu, Xinrong Ma, Ling Dang, Yuelan Zhang, Furong Gou, Xin Tian

**Affiliations:** 1 School of Nursing, Ningxia Medical University, Yinchuan, China; 2 Department of Psychiatry and Clinical Psychology, Ningxia Ning-An Hospital, Ningxia Mental Health Center, Yinchuan, China; 3 Department of Emergency Medicine, Shanxi Bethune Hospital, Shanxi Academy of Medical Sciences, Third Hospital of Shanxi Medical University, Tongji Shanxi Hospital, Taiyuan, Shanxi, China

**Keywords:** adolescents with depressive disorder, depressive symptoms, excessive Internet use, sleep quality, social anxiety

## Abstract

**Background:**

Adolescents with depressive disorders are at elevated risk for adverse mental health outcomes, and excessive Internet use has been increasingly linked to greater symptom severity. Therefore, this study aimed to examine the chain mediating roles of social anxiety and sleep quality in the association between excessive Internet use and depressive symptoms among adolescents with depressive disorders.

**Methods:**

A cross-sectional design was used. A total of 266 Chinese adolescents with clinically diagnosed depressive disorders (M = 15.79 years, SD = 1.85; 71.4% female) were assessed using the Internet Addiction Test, Zung Self-Rating Depression Scale, Social Anxiety Scale for Children, and Pittsburgh Sleep Quality Index. Correlation analyses and bootstrapping methods were conducted using SPSS and the PROCESS macro to examine the chain mediating effects of social anxiety and sleep quality.

**Results:**

The total indirect effect of excessive Internet use on depressive symptoms accounted for 65.66% of the total effect. Specifically, the indirect effects via social anxiety and sleep quality accounted for 24.10% and 26.51% of the total effect, respectively. In addition, the chain mediating effect of social anxiety and sleep quality was significant, accounting for 14.76% of the total effect.

**Conclusion:**

Excessive Internet use was positively associated with more severe depressive symptoms among adolescents with depressive disorders, both directly and indirectly through the chain mediating effects of social anxiety and sleep quality. These findings highlight potential targets for preventing and intervening in excessive Internet use among this population.

## 1 Introduction

Adolescence is a critical developmental stage characterized by rapid biological, cognitive, and psychological changes (1). It is also a period of heightened vulnerability to mental health disorders, particularly depressive disorder (2). Core features of depressive disorder include persistent low mood, diminished interest or pleasure in daily activities, and physiological disturbances such as sleep disruption and appetite changes (3). Globally, the prevalence of depressive disorder among adolescents varies across regions, with some estimates reaching as high as 37% (4). In China, *the Report on the Development of National Mental Health* (2021–2022) indicates that the detection rate of depressive symptoms among adolescents is 14.8%. The World Health Organization (WHO) has identified adolescent depression as a leading cause of disability worldwide and a major public health concern (5). Adolescent depression is also associated with a range of adverse outcomes, including poorer academic performance, social withdrawal, and increased risk of self-harm and suicide (6, 7). Despite substantial progress in identifying biological and psychosocial risk factors (8), the mechanisms linking contemporary digital behaviors to adolescent mental health remain insufficiently understood. Therefore, further research is needed to explore potential contributors to depressive symptoms in adolescents within the context of contemporary digital behaviors.

Internet use encompasses a range of activities through which individuals access online resources to meet their needs via devices such as smartphones, tablets, and computers (9). In contrast, excessive Internet use is characterized by difficulty voluntarily reducing usage time, along with psychological distress (e.g., anxiety or depressive symptoms) or physical discomfort when access is restricted (10). In China, Internet penetration among adolescents reached 97.2% in 2022, approaching saturation. Internet use is also occurring at younger ages, with approximately 90% of Chinese adolescents owning personal Internet-enabled devices and primarily accessing the Internet via smartphones (11). While the Internet facilitates learning, social interaction, and entertainment, excessive use has been linked to adverse psychological outcomes, including elevated depressive symptoms (12). Among adolescents with depressive disorders, this association may be particularly complex. Prior research suggests that the relationship between Internet use and depressive symptoms may be bidirectional, whereby excessive Internet use is associated with higher levels of depressive symptoms, while adolescents with more severe depressive symptoms may also be more likely to engage in Internet use as a coping strategy or due to reduced offline engagement (13). Some studies have reported that excessive screen time, especially passive engagement with social media, is associated with higher levels of depressive symptoms (14, 15). In contrast, other studies suggest that Internet use may compensate for limited offline social support among individuals with depression, which is associated with lower levels of depressive symptoms (16, 17). These mixed findings highlight the need for further investigation into the associations linking Internet use and depressive symptoms, particularly among vulnerable adolescent populations.

Social anxiety may function as a mediating variable in this relationship. It is characterized by fear of negative evaluation and avoidance of social interactions (18–20), and commonly co-occurs with depressive disorder, sharing underlying neurobiological and cognitive vulnerabilities (21). Previous research indicates that higher levels of social anxiety are associated with greater depressive symptom severity (22). Notably, the relationship between social anxiety and depressive symptoms may also be bidirectional, with each potentially reinforcing the other over time (23). A decade-long longitudinal study found that social anxiety was associated with a significantly increased risk of subsequent depressive disorder (24). In a study by Moscovitch, multilevel mediation analysis showed that improvements in social anxiety predicted 91% of subsequent improvements in depressive symptoms (25). The relationship between Internet use and social anxiety may also be bidirectional. Compared with face-to-face interactions, engagement with online social media may replace aspects of real-world socialization, which may be associated with reduced offline social support and higher levels of social anxiety (26). Platforms characterized by curated self-presentation (e.g., Facebook, TikTok) are also associated with increased concerns about negative evaluation, particularly among adolescents predisposed to negative self-evaluation (27). Moreover, individuals with higher levels of social anxiety tend to engage more frequently in online interactions to meet their social needs (28). Among adolescents with depressive disorder, social withdrawal may function both as a symptom and as a coping strategy. Internet-related social experiences may coincide with increased feelings of isolation and hopelessness, which are associated with more severe depressive symptoms (29).

Sleep quality represents another important factor in this association. Sleep disturbance is a common and clinically significant symptom among adolescents with depressive disorder, with surveys reporting sleep problems in up to 87% of this population (30). Existing research suggests that poor sleep quality is not only a core feature of depressive disorder but may also play a role in its course (31). Importantly, the relationship between sleep quality and depressive symptoms is widely considered to be bidirectional (32). Poor sleep quality may exacerbate depressive symptoms, whereas depressive symptoms may also impair sleep quality. Meaklim et al. found that insomnia was significantly associated with an increased risk of depressive symptoms over longitudinal follow-up (33). Poor sleep quality has been associated with greater depressive symptom severity, potentially through mechanisms such as impaired mood regulation, increased irritability, and reduced stress tolerance (34). Recent evidence suggests that Internet use may be associated with disruptions in circadian rhythms and sleep patterns (35). Specifically, exposure to blue light at night has been linked to suppressed melatonin secretion, delayed sleep onset, and reduced slow-wave sleep (36). In addition, frequent checking of notifications or engagement in stimulating online activities (e.g., gaming) may be associated with heightened cognitive arousal and poorer sleep quality (37). Among adolescents with depressive disorder, whose sleep–wake cycles are often dysregulated, excessive Internet use may be associated with the maintenance of depressive symptoms.

Taken together, the existing literature suggests that social anxiety and sleep quality play important roles in the association between excessive Internet use and depressive symptoms. From a theoretical perspective, the broaden-and-build theory of positive emotions provides a useful framework for understanding these relationships. Proposed by Fredrickson, this theory posits that positive emotions broaden individuals’ momentary thought–action repertoires and contribute to the development of enduring personal resources, including social support and physiological resilience (38). In contrast, negative emotional states, such as social anxiety, may constrain coping strategies and reduce individuals’ capacity to manage stress effectively. These processes may be associated with the depletion of psychological and physiological resources, including sleep quality. Among adolescents with depressive disorders, excessive Internet use may be associated with higher levels of social anxiety, potentially reflecting increased negative social comparisons and heightened sensitivity to external evaluation (39). Elevated social anxiety may also be associated with increased physiological arousal and cognitive rumination, which may be linked to poorer sleep quality (40). Poor sleep quality may further be associated with reduced emotional regulation capacity and resilience, and with more severe depressive symptoms (41). Thus, the broaden-and-build theory supports not only the potential independent mediating roles of social anxiety and sleep quality but also their possible chain mediating pathway in the association between excessive Internet use and depressive symptoms.

Therefore, this study aimed to examine the associations and chain mediating roles of excessive Internet use, social anxiety, and sleep quality in relation to depressive symptoms among adolescents with depressive disorder. Based on the broaden-and-build theory of positive emotions, the following hypotheses were proposed:

H1: Excessive Internet use is positively associated with depressive symptoms in adolescents with depressive disorders.
H2: Social anxiety mediates the association between excessive Internet use and depressive symptoms.
H3: Sleep quality mediates the association between excessive Internet use and depressive symptoms.
H4: Social anxiety and sleep quality serve as chain mediators between excessive Internet use and depressive symptoms in adolescents with depressive disorders.

**Figure 1:**
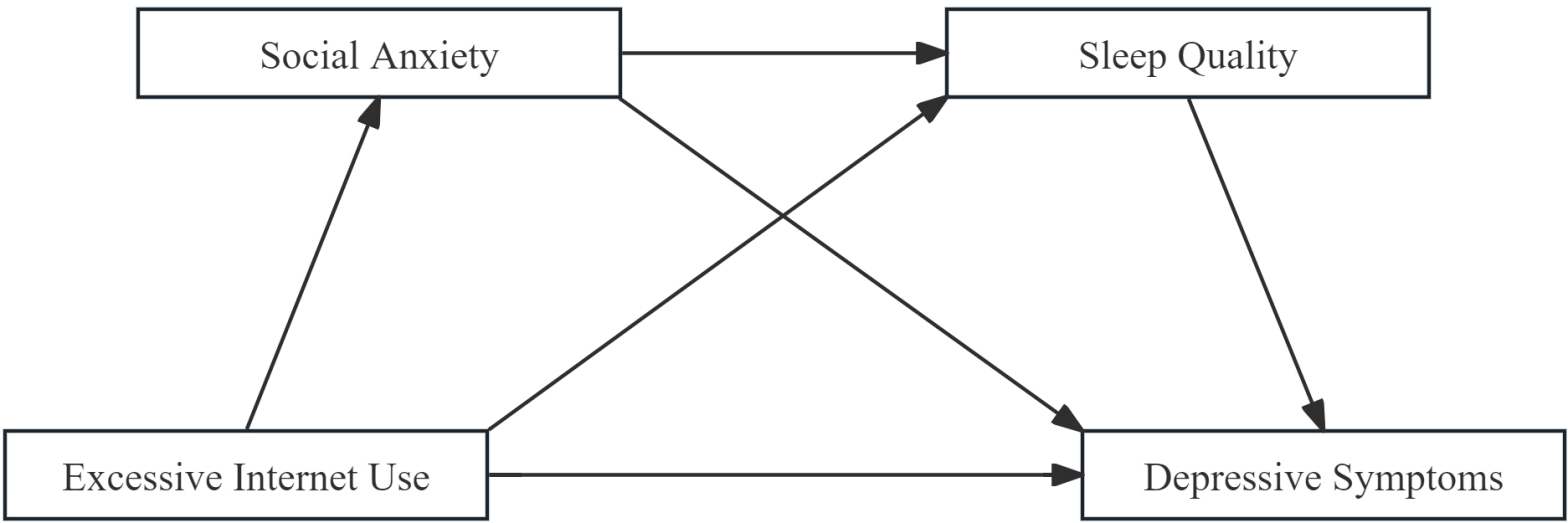
The chain mediation model of Internet use and depressive symptoms in adolescents with depressive disorders.

Based on these hypotheses, a chain mediation model was constructed, as shown in **Figure 1**. The goal of this study is to better understand the mechanisms underlying depressive symptoms among adolescents and to identify key targets for intervention.

## 2 Method

### 2.1 Study design and participants

This study employed a cross-sectional design and convenience sampling method. Data were collected from February to December 2024 in the outpatient and inpatient departments of Ning’an Hospital, Ningxia, China. According to the World Health Organization’s definition of adolescence (10–19 years) (42), participants were required to meet the following inclusion criteria: (1) a diagnosis of depressive disorder based on the Diagnostic and Statistical Manual of Mental Disorders, Fifth Edition (DSM-5), confirmed by a psychiatrist; (2) aged between 10 and 19 years; and (3) a stable clinical condition with consistent medication type and dosage. The exclusion criteria included (1) the presence of severe comorbid physical illnesses and (2) reading disabilities. All inpatient participants were admitted to the psychiatric ward for the treatment of depressive disorder rather than for other medical conditions. The study was conducted by a research team consisting of one chief psychiatrist and four graduate students, all of whom received standardized training prior to data collection. Participants were recruited through physician referrals as well as posters displayed in outpatient clinics and inpatient wards. After obtaining written informed consent from both participants and their legal guardians, all participants independently completed an online questionnaire via the Wenjuanxing platform (https://www.wjx.cn) in a quiet room within the hospital. The average time required to complete all measures was approximately 20 to 25 minutes. A total of 266 adolescents with depressive disorder were included in the final analysis. According to the recommendations of Fritz and MacKinnon (43), a sample size of at least 200 participants is required to detect small-to-medium mediation effects with adequate statistical power. Therefore, the sample size in this study met the recommended criteria for multivariable mediation analysis. The study was approved by the Ethics Committee of Ning’an Hospital (Approval No.: 2023-WS-022) and was conducted in accordance with the Declaration of Helsinki.

To minimize response bias, all questionnaires were completed anonymously, and participants were assured that their responses would remain confidential and be used solely for research purposes. Although mediation analysis typically assumes a temporal sequence among variables, the present study employed a cross-sectional design due to feasibility and practical considerations. Nevertheless, the chain mediation model was grounded in established theoretical frameworks (e.g., the broaden-and-build theory) and prior empirical evidence supporting the associations among excessive Internet use, social anxiety, sleep quality, and depressive symptoms.

### 2.2 Measurement

#### 2.2.1 Demographic characteristics

Demographic characteristics were collected using a self-administered questionnaire, including gender (male or female), age, only-child status (only child or with siblings), years of education, and whether receiving pharmacotherapy (yes or no).

#### 2.2.2 Excessive Internet use

Excessive Internet use was assessed using the Internet Addiction Test (IAT), developed by Young to evaluate Internet use behaviors among adolescents (44). The IAT is a one-dimensional scale consisting of 20 items rated on a five-point Likert scale (1–5), with total scores ranging from 20 to 100; higher scores indicate more severe Internet addiction. An example item is: “How often do you find that you stay online longer than you intended?” The IAT has demonstrated good reliability and validity among Chinese adolescents (45). In the present study, the Cronbach’s alpha coefficient was 0.942.

#### 2.2.3 Depressive symptoms

Depressive symptoms were assessed using the Zung Self-Rating Depression Scale (SDS) (46). The scale consists of 20 items rated on a four-point Likert scale (1–4), with 10 items reverse scored. The sum of all items constitutes the raw SDS score. The standardized score is calculated by multiplying the raw total score by 1.25, resulting in a score ranging from 25 to 100; higher scores indicate more severe depressive symptoms. An example item is: “I feel downhearted and blue.” Previous studies have demonstrated that the SDS has good reliability and validity among Chinese adolescents (47). In this study, the Cronbach’s alpha coefficient was 0.887.

#### 2.2.4 Social anxiety

Social anxiety was assessed using the Social Anxiety Scale for Children (SASC), developed by La Greca (48). The scale consists of 10 items divided into two dimensions. Each item is rated on a three-point scale (0–2), yielding a total score ranging from 0 to 20; higher scores indicate greater social anxiety. An example item is: “I’m worried about being made fun of.” The Chinese version of the SASC has been widely used and validated among adolescents (49, 50). In this study, the Cronbach’s alpha coefficient was 0.959.

#### 2.2.5 Sleep quality

Sleep quality was assessed using the Pittsburgh Sleep Quality Index (PSQI), developed by Buysse et al. (51). The PSQI evaluates sleep quality over the past month and consists of 18 items across seven components. Each component is scored from 0 to 3, and the sum of these component scores yields a global score ranging from 0 to 21; higher scores indicate poorer sleep quality. Previous studies have demonstrated that the PSQI is suitable for assessing sleep quality among Chinese adolescents (52). In this study, the Cronbach’s alpha coefficient was 0.764.

### 2.3 Statistical analysis

All statistical analyses were performed using SPSS version 26.0. First, data screening and descriptive analyses were conducted. Normality was assessed using the Shapiro–Wilk test and visual inspection of histograms and Q–Q plots. Continuous variables were expressed as mean ± standard deviation (SD) when normally distributed and as median and interquartile range (IQR) when not normally distributed, while categorical variables were presented as frequencies and percentages [n (%)]. Second, group comparisons and correlation analyses were performed. Group differences in study variables across gender, only-child status, and pharmacotherapy status were examined using independent samples t-tests or Mann–Whitney U tests, depending on normality assumptions. Correlation analyses were conducted among age, years of education, excessive Internet use, social anxiety, sleep quality, and depressive symptoms. Pearson correlation coefficients were used for normally distributed variables, whereas Spearman correlation coefficients were applied when assumptions of normality were not satisfied. Third, assumption checking and bias assessment were conducted. Common method bias was assessed using Harman’s single-factor test. The results showed that 11 factors had eigenvalues greater than 1, with the first factor accounting for 28.62% of the total variance, below the threshold of 40%, indicating no serious common method bias. Multicollinearity was assessed using variance inflation factors (VIFs), all of which were below 5, indicating no multicollinearity issues. Finally, mediation analysis was performed. A chain mediation model was constructed using the PROCESS macro version 4.1 for SPSS (Model 6). Indirect effects were estimated using 5,000 bootstrap samples with 95% confidence intervals (CIs). Statistical significance was set at p < 0.05. Gender and only-child status were included as covariates due to their significant associations with key study variables, whereas pharmacotherapy status was excluded based on non-significant preliminary results.

## 3 Results

### 3.1 Demographic characteristics

**Table 1** presents the demographic characteristics of the participants and the differences in key variables across demographic groups. A total of 266 adolescents with depressive disorder were included in this study, with females accounting for 71.4% of the sample. The mean age of participants was 15.79 years (SD = 1.85), and the median age was 16 years. Additionally, 80.8% of participants had siblings. The mean number of years of education was 9.57 (SD = 1.82). Moreover, 81.1% of participants were receiving pharmacotherapy. Significant differences were found in depressive symptoms, social anxiety, and sleep quality scores between genders (*p* < 0.05). Social anxiety scores also differed significantly across only-child status groups (*t* = 2.669, *p* < 0.01). Therefore, these variables were statistically controlled in all subsequent mediation models to minimize potential confounding effects. **Table 1** also lists the means and standard deviations of key variables across different demographic subgroups.

**Table 1:** The differences among sample characteristics, excessive internet use, depressive symptoms, social anxiety, and sleep quality (N = 266).

Variable	M (SD)/*N*(%)	Excessive internet use		Depressive symptoms		Social anxiety		Sleep quality	
		M (SD)	*t/r*	M (SD)	*t/r*	M (SD)	*t/r*	M (SD)	*t/r*
Gender			0.235		-2.681^**^		-2.610^*^		-2.280^*^
Male	76(28.6)	58.75(13.31)		61.93(15.36)		12.22(8.53)		8.83(4.58)	
Female	190(71.4)	58.11(15.12)		67.25(14.31)		15.07(6.62)		10.26(4.62)	
Age	15.79(1.85)		-0.079		-0.008		-0.016		0.077
Only-child Status			-0.746		-0.435		2.669^**^		1.122
Only Child	51(19.2)	56.62(17.99)		64.92(14.66)		16.69(7.42)		10.51(4.74)	
With Siblings	215(80.8)	58.62(13.70)		65.93(14.84)		13.68(7.19)		9.70(4.62)	
Years of Education	9.57(1.82)		0.053		0.083		0.057		0.110
Pharmacotherapy			-1.072		0.501		0.379		-1.321
Yes	179(67.3)	57.57(15.30)		66.05(15.11)		14.37(7.18)		9.59(4.49)	
No	87(32.7)	59.61(13.04)		65.08(14.16)		14.01(7.62)		10.39(4.93)	

^*^ *p* < 0.05, ^**^ *p* < 0.01, ^***^ *p* < 0.001.

M, mean; SD, standard deviation.

### 3.2 Correlation between variables

**Table 2** shows the correlations of the key study variables. All independent variables were statistically significantly associated with depressive symptoms. Specifically, excessive Internet use (*r* = 0.326, *p* < 0.001), social anxiety (*r* = 0.478, *p* < 0.001) and sleep quality (*r* = 0.566, *p* < 0.001) were positively correlated with depressive symptoms; social anxiety (*r* = 0.295, *p* < 0.001) and sleep quality (*r* = 0.328, *p* < 0.001) were positively correlated with excessive Internet use; social anxiety was positively correlated with sleep quality (*r* = 0.467, *p* < 0.001). These correlation results provide preliminary empirical support for the hypothesized associations among study variables.

**Table 2:** Correlations among the measured variables (*N* = 266).

Variables	M	SD	1	2	3	4
1.Excessive Internet Use	58.24	14.60	1			
2.Depressive Symptoms	65.73	14.79	0.326^***^	1		
3.Social Anxiety	14.26	7.32	0.295^***^	0.478^***^	1	
4.Sleep Quality	9.85	4.65	0.328^***^	0.566^***^	0.467^***^	1

^*^ *p* < 0.05, ^**^ *p* < 0.01, ^***^ *p* < 0.001.

M, mean; SD, standard deviation.

### 3.3 Chained mediation analysis

**Figure 2:**
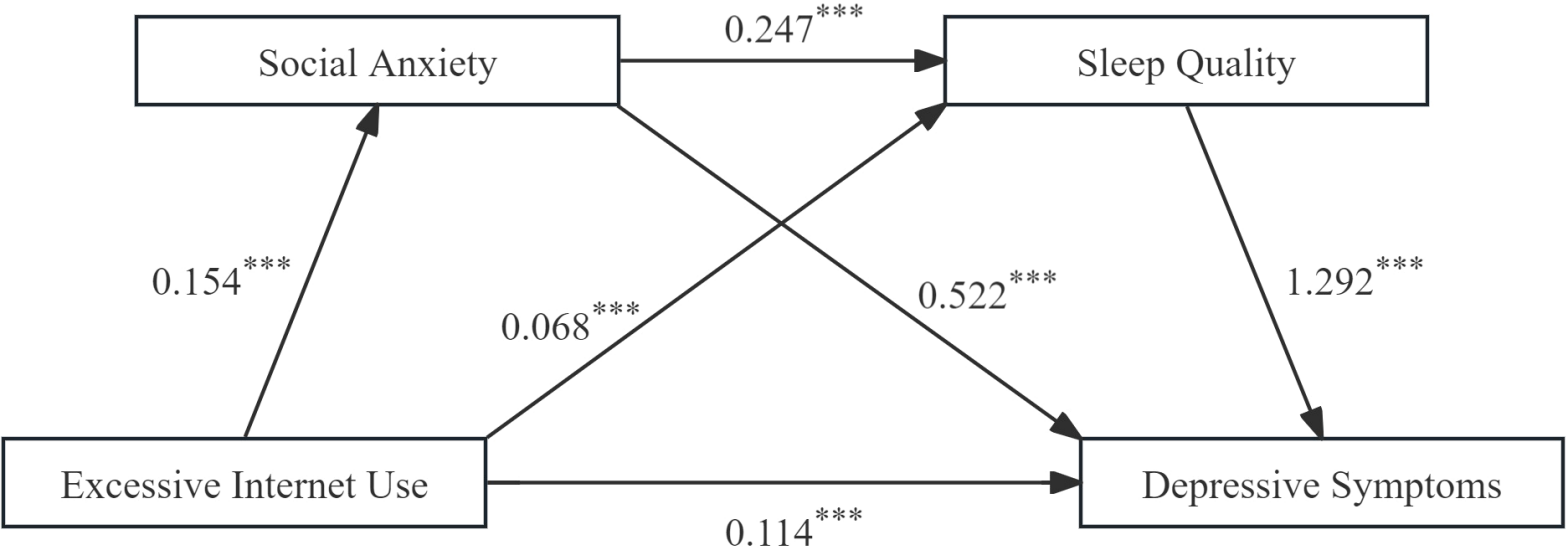
The chain mediation model and the B of each path. *p < 0.05, **p < 0.01, ***p< 0.001.

**Table 3** presents the regression results for the chained mediated effects model. **Figure 2** depicts the coefficients and significance of each path. After controlling for gender and only-child status, excessive Internet use was significantly associated with depressive symptoms in adolescents with depressive disorder (*B* = 0.332, *p* < 0.001; total effect) and remained significantly associated in the direct effect model (*B* = 0.114, *p* < 0.05). Excessive Internet use was positively associated with social anxiety (*B* = 0.154, *p* < 0.001) and sleep quality (*B* = 0.068, *p* < 0.01). Social anxiety was positively associated with sleep quality (*B* = 0.247, *p* < 0.001) and depressive symptoms (*B* = 0.522, *p* < 0.001). Sleep quality was positively associated with depressive symptoms in adolescents with depressive disorder (*B* = 1.292, *p* < 0.001).

**Table 3:** Regression result of the chain mediating effect model (*N* = 266).

Outcome variable	Predictive variable	*R^2^*	*F*	*B*	*SE*	*t*
Equation 1		0.151	15.477			
Social Anxiety	Excessive Internet Use			0.154	0.029	5.384^***^
	Gender			2.882	0.921	3.131^**^
	Only-child Status			3.276	1.058	3.097^**^
Equation 2		0.263	23.309			
Sleep Quality	Excessive Internet Use			0.068	0.018	3.838^***^
	Social Anxiety			0.247	0.037	6.731^***^
	Gender			0.755	0.556	1.359
	Only-child Status			0.197	0.638	0.309
Equation 3		0.403	35.096			
Depressive Symptoms	Excessive Internet Use			0.114	0.053	2.171^*^
	Social Anxiety			0.522	0.114	4.587^***^
	Sleep Quality			1.292	0.178	7.273^***^
	Gender			2.079	1.600	1.299
	Only-child Status			-3.422	1.832	-1.868
Equation 4		0.135	13.578			
Depressive Symptoms	Excessive Internet Use			0.332	0.058	5.697^***^
	Gender			5.478	1.878	2.917^**^
	Only-child Status			-1.414	2.158	-0.192

All estimated coefficients are unstandardized. Adjusted gender and only-child status, ^*^ *p* < 0.05, ^**^ *p* < 0.01, ^***^ *p* < 0.001.

**Table 4** presents the overall, direct, and indirect effects. A bootstrap method with 5000 sample tests was used to assess that none of the 95% confidence intervals for the three mediating pathways contained zero, and therefore a mediating effect was present. The direct effect value of excessive Internet use on depressive symptoms was 0.114. In addition, the effect value of excessive Internet use on depressive symptoms through social anxiety was 0.080, with a mediated effect of 24.10%. The effect value of excessive Internet use on depressive symptoms through sleep quality was 0.088, with a mediated effect of 26.51%. The effect value of internet use on depressive symptoms through social anxiety and sleep quality was 0.049, which is 14.76% of the total effect. As shown in **Table 4**, the total mediated effect of excessive Internet use on depressive symptoms was 65.66%.

**Table 4:** Result of chain mediating effect (*N* = 266).

	Effect	Boot SE	Boot LLCI	Boot ULCI	Ratio of indirect to total effect
Total effect	0.332	0.058	0.217	0.447	—
Direct effect	0.114	0.053	0.011	0.218	—
Total indirect effect	0.218	0.035	0.152	0.289	65.66%
Excessive Internet Use→social Anxiety→Depressive Symptoms	0.080	0.025	0.037	0.133	24.10%
Excessive Internet Use→sleep Quality→Depressive Symptoms	0.088	0.024	0.044	0.137	26.51%
Excessive Internet Use→social Anxiety→sleep Quality→Depressive Symptoms	0.049	0.014	0.025	0.079	14.76%

## 4 Discussion

The present study found that excessive Internet use was significantly and positively associated with depressive symptoms among adolescents with depressive disorders, such that higher levels of Internet use corresponded to greater depressive symptom severity. Accordingly, H1 was supported. This finding is consistent with previous research. For example, Boers reported that each additional hour of average Internet use was associated with a 0.69-unit increase in depressive symptom severity (53). In addition, a systematic narrative review by Saleem et al., synthesizing evidence from 67 studies, indicated that both problematic social media use and higher levels of use were associated with depressive symptoms in children and adolescents (54). Together, these findings support a robust association between excessive Internet use and less favorable mental health outcomes. From a theoretical perspective, this association may be partially interpreted within the framework of cognitive–behavioral theory (55), which emphasizes the role of cognitive patterns and behavioral tendencies in shaping emotional experiences. Among adolescents with depressive disorders, frequent reliance on online engagement—particularly social media use—may coincide with negative automatic thoughts (e.g., “I am not good enough”) and avoidance of offline social interactions. These patterns may be related to increased feelings of isolation and diminished self-worth. Importantly, the Internet itself is not inherently harmful; rather, its psychological correlates appear to depend on how it is used. Interventions in this population may benefit from focusing on promoting purposeful and constructive digital engagement, such as participation in online mutual support communities or the use of positive psychology–based applications. Accordingly, intervention strategies may emphasize fostering intentional and reflective Internet use, incorporating digital literacy education, and integrating cognitive–behavioral approaches to mitigate the negative associations between excessive Internet use and depressive symptoms.

The present study found that, among adolescents with depressive disorders, social anxiety served as a significant mediator in the association between excessive Internet use and depressive symptoms. Higher levels of Internet use were associated with higher levels of social anxiety, which were also associated with more severe depressive symptoms. Accordingly, H2 was supported. Regarding the association between excessive Internet use and social anxiety, a nationally representative cross-sectional survey of Finnish students aged 14–18 years conducted by Kajastus reported that social anxiety was associated with excessive Internet use (56), which is consistent with the present findings. Other studies have similarly documented significant associations between digital addiction and levels of social anxiety (57). In addition, prior research indicates that social anxiety is strongly associated with depressive symptoms (23). According to cognitive models of anxiety (58), individuals with social anxiety tend to avoid offline interactions due to heightened concerns about negative evaluation and may instead engage more frequently in online environments. Such patterns may be associated with short-term relief from distress while also coinciding with increased social avoidance and negative self-appraisals, which are linked to higher levels of depressive symptoms. From a developmental psychology perspective (59), adolescence represents a critical period for the formation of social bonds and self-identity. Greater reliance on online interactions in place of offline engagement may be associated with disruptions in these developmental processes, with potential implications for mental well-being. Interventions in this context may benefit from not only addressing patterns of Internet use but also considering underlying social anxiety. Incorporating exposure-based approaches or structured social skills training into school-based or clinical programs may support adolescents in gradually engaging in offline social interactions, strengthening interpersonal connections, and potentially alleviating the maladaptive pattern linking excessive Internet use, social anxiety, and depressive symptoms.

The present study found that sleep quality served as a significant mediator in the association between excessive Internet use and depressive symptoms. Higher levels of Internet use were associated with poorer sleep quality, which was also associated with more severe depressive symptoms. Accordingly, H3 was supported. Consistent with the present findings, previous research has examined the roles of social support and sleep quality in the relationship between Internet use and depression among college students and has reported that sleep quality mediates the association between excessive Internet use and depressive symptoms (60). Excessive Internet use may reflect prolonged time spent online, which may coincide with alterations in sleep–wake patterns and is associated with sleep disturbances (61). Prior studies have also indicated that sleep disturbances are associated with depressive symptoms (62). From a theoretical perspective, this pattern may be interpreted within the framework of the emotion regulation model (63), which emphasizes the role of sleep in maintaining emotional stability. Poor or insufficient sleep is associated with altered prefrontal functioning, heightened emotional reactivity, and increased vulnerability to depressive symptoms. In addition, nighttime screen exposure has been linked to reduced melatonin secretion, delayed sleep onset, and disrupted sleep architecture, which may be particularly relevant for adolescents with depressive disorders (36). Interventions in this context may benefit from incorporating sleep-focused behavioral strategies, such as promoting regular sleep routines, limiting electronic device use before bedtime, and implementing cognitive–behavioral approaches to sleep management.

The present study further identified that social anxiety and sleep quality jointly formed a significant chain mediating pathway linking excessive Internet use to depressive symptoms, supporting H4. This suggests that excessive Internet use may not only be associated with depressive symptoms directly or through single mediating pathways but may also be associated with depressive symptoms through a sequential mechanism: higher levels of social anxiety are associated with poorer sleep quality, which is in turn associated with more severe depressive symptoms. Previous findings have shown that social anxiety is positively associated with depressive symptoms and poor sleep quality, and that social anxiety is indirectly associated with depressive symptoms through sleep quality, which is consistent with the findings of the present study (64). Excessive Internet use may be associated with increased social anxiety among adolescents by reducing real-life social interactions, triggering social comparisons, or encouraging reliance on online socialization. According to the broaden-and-build theory of positive emotions (38), social anxiety may further be associated with emotional stress and excessive worry, which may interfere with sleep onset and be associated with poorer sleep quality. Reduced sleep quality, in turn, is associated with a weakened physiological basis of emotion regulation and is associated with higher levels of depressive symptoms. This pathway reveals a complex mechanism linking excessive Internet use and depressive symptoms. Therefore, intervention strategies should adopt an integrative approach targeting both psychological and physiological domains. Alleviating social anxiety through exposure-based therapy or group social skills training, combined with sleep hygiene interventions, may enhance the overall effectiveness of adolescent depression treatment programs.

## 5 Limitations and prospects

Despite its valuable contributions, this study has several limitations that should be acknowledged. First, it should be noted that the relationships among excessive Internet use, depressive symptoms, social anxiety, and sleep quality may be bidirectional. Given the cross-sectional design, the temporal ordering and causal direction of these associations cannot be determined. Therefore, although the sequential mediation model was theoretically grounded, it should be interpreted as reflecting statistical associations rather than causal pathways. Future longitudinal or experimental studies are needed to clarify temporal relationships and further examine the proposed mediation pathways. Second, participants ranged in age from 10 to 19 years, spanning middle school to university students. This relatively wide age range involves substantial developmental variability, which may influence the stability and generalizability of the findings. Future research could adopt age-stratified sampling or analytic approaches to reduce the impact of developmental heterogeneity and allow for more targeted comparisons across developmental stages. Third, the use of convenience sampling from a single hospital setting may introduce selection bias and limit the representativeness of the findings. Future studies could employ multi-center designs and more rigorous sampling strategies, including recruitment from medical institutions, communities, and schools, to enhance generalizability. Fourth, all variables were assessed using self-report measures, which may be subject to response biases such as social desirability and recall bias. In addition, self-report data from younger adolescents may be less reliable due to differences in cognitive development and comprehension. Future research could incorporate multi-informant approaches (e.g., parent or teacher reports) and objective measures (e.g., actigraphy for sleep) to improve measurement validity. Finally, although several demographic variables were controlled as covariates, other potential confounding factors (e.g., family environment, academic pressure) were not assessed and may have influenced the observed associations. Future studies should include a broader range of contextual and family-related variables to better capture the complexity of adolescent mental health.

## 6 Conclusion

The findings of this study suggest that excessive Internet use is associated with more severe depressive symptoms among adolescents with depressive disorders. In addition, bootstrapping analyses supported a sequential mediation model in which social anxiety and sleep quality sequentially mediated the association between Internet use and depressive symptoms. Overall, the proposed theoretical model was supported in the present sample. Future longitudinal studies are warranted to clarify temporal ordering and to examine the potential causal direction of these mediation pathways.

## Data Availability

The raw data supporting the conclusions of this article will be made available by the authors, without undue reservation.
